# Airway Intervention in Paediatric Angioedema: A National Analysis of Clinical Predictors and Outcomes

**DOI:** 10.1111/coa.70127

**Published:** 2026-05-31

**Authors:** Saharsh Talwar, Aryan Mahajan, Gaurav N. Pathak, Babar K. Rao, Rachel Kaye

**Affiliations:** ^1^ Rutgers Robert Wood Johnson Medical School Piscataway New Jersey USA; ^2^ Department of Dermatology Weill Cornell School of Medicine New York New York USA; ^3^ Department of Otolaryngology–Head and Neck Surgery Rutgers New Jersey Medical School Newark New Jersey USA

**Keywords:** airway intervention, angioedema, health disparities, KID, paediatric angioedema

## Abstract

**Objective:**

To characterize paediatric hospitalizations for angioedema and identify the prevalence and outcomes of airway interventions using a large national database.

**Methods:**

We performed a cross‐sectional analysis of the Kids' Inpatient Database (2003–2019), including paediatric patients (0–20 years) hospitalized with angioedema identified by ICD‐9/10 codes. Outcomes included hospital length of stay (LOS), total charges, and in‐hospital mortality. Multivariable logistic regression evaluated predictors of airway intervention, and comparative analyses assessed outcome differences between patients with and without airway procedures.

**Results:**

Among 4238 paediatric angioedema hospitalizations, 174 (4.1%) underwent airway procedures. Compared to non‐intervention cases, these patients had significantly longer LOS (median 6 vs. 2 days, *p* < 0.001), higher charges ($59 571 vs. $7628, *p* < 0.001), and higher mortality (5.8% vs. 0.15%, *p* < 0.001). Black (adjusted odds ratio [AOR] 5.23, 95% CI 1.56–19.30) and Hispanic race (AOR 7.83, 95% CI 1.51–44.89) were associated with higher odds of intervention, while hospitalizations in the Northeast (AOR 0.10, 95% CI 0.015–0.55) and South (AOR 0.13, 95% CI 0.03–0.49) had lower odds compared to the Midwest.

**Conclusion:**

Airway interventions in paediatric angioedema are rare but linked to significantly higher morbidity, mortality, and healthcare costs. Geographic and racial disparities highlight the need to explore systemic factors shaping airway management decisions.

## Key Points

- Study: Analyzed ~4,238 pediatric angioedema hospitalizations (KID, 2003–2019) to identify predictors and outcomes of airway intervention.
- Main findings: 4.1% needed airway procedures. Those patients had 3× longer stays, 8× higher charges, and ~40× higher mortality compared to non‐intervention cases. Tracheostomy cases were the most severe (23.5% mortality, $290K median charges).
- Disparities: Black (AOR 5.23) and Hispanic (AOR 7.83) patients had significantly higher odds of intervention. Northeast and South regions had lower odds compared to the Midwest.
- Comorbidity clue: Intervention patients had lower rates of asthma, urticaria, and anaphylaxis—suggesting more bradykinin‐mediated (harder to treat) cases rather than histaminergic ones.

## 1 Introduction

Angioedema is an immunologic presentation characterized by acute, localized swelling of the skin and mucous membranes. While often presenting with cutaneous manifestations, upper airway involvement can rapidly progress to respiratory compromise [1]. Edema results from increased vascular permeability [2].

In paediatric patients, angioedema may arise from idiopathic causes, allergic or drug‐induced reactions, and genetic conditions. A key distinction for clinical management of angioedema lies in differentiating between mast cell‐mediated and bradykinin‐mediated forms of angioedema, as their underlying pathophysiologies dictate vastly different treatment approaches [3].

Mast cell‐mediated, or histaminergic, angioedema is commonly associated with urticaria (hives) and pruritus, and it may occur as part of an anaphylactic reaction [3]. This type typically responds well to standard treatments, including antihistamines and epinephrine [4]. Common triggers for mast cell‐mediated angioedema include certain foods, medications (such as non‐steroidal anti‐inflammatory drugs), and insect stings [3]. Conversely, bradykinin‐mediated angioedema is characterized by isolated, non‐pruritic, non‐pitting swelling that is notably refractory to antihistamines and epinephrine. Hereditary angioedema (HAE) is a prominent example of this form, stemming from a deficiency or dysfunction of the C1 inhibitor protein, which leads to uncontrolled production of bradykinin [5]. Another common example of bradykinin‐mediated angioedema is the angiotensin‐converting enzyme (ACE) inhibitor‐induced subtype, which is significantly prevalent in adults as it comprises approximately 30% of all adult angioedema episodes in emergency departments [6].

In all types of angioedema the clinical concern is acute upper airway swelling, encompassing pharyngeal, laryngeal, and oral cavity edema. Such edema can rapidly progress to respiratory distress and potentially fatal asphyxiation, necessitating urgent intervention [7]. Historical reports suggest that untreated hereditary angioedema involving the upper airway has been associated with mortality rates ranging from 30% to 50% [8]. In children, the risk of rapid asphyxia is amplified due to their smaller airway diameters, making prompt recognition and management even more crucial [9].

The inherent potential for rapid airway compromise and severe implications of airway involvement in angioedema demand study to prevent such complications. In particular, the paediatric angioedema population necessitates further evaluation of the factors impacting critical airway management given it's an at‐risk population. Existing epidemiological data on angioedema‐related emergency department visits and hospitalizations are often hampered by a lack of clarity in case definitions and a reliance on broad International Classification of Diseases (ICD) codes, which can obscure the true incidence of these severe events. In this study, we aim to both evaluate the impact of airway intervention for paediatric angioedema and to determine clinical predictors of intervention in these patients.

## 2 Materials and Methods

We conducted a cross‐sectional analysis of the Kids' Inpatient Database (KID) to evaluate intubation in paediatric angioedema. KID is the largest publicly available U.S. paediatric inpatient database, providing national estimates of hospital inpatient stays for patients younger than 21 years of age. Data from 2003, 2006, 2009, 2012, 2016, and 2019 were included, representing KID's triennial releases.

The study population consisted of children aged 0–20 years who were hospitalized with a primary or secondary diagnosis of angioedema. Angioedema was identified using specific ICD codes. For ICD‐9, codes 995.1 (Angioneurotic edema) and 277.6 (Hereditary angioedema) were utilized. For ICD‐10, codes T78.3XXA, T78.3XXD, and T78.3XXS (Angioneurotic edema), along with D84 (C1 inhibitor deficiency), were employed.

The primary outcome was airway intervention, defined as intubation or tracheostomy. ICD‐9 Procedure Codes included: Intubation (96.04, 96.05) and Tracheostomy (31.1x–31.2x). ICD‐10 Procedure Codes included: Intubation (0BH17EZ, 5A1935Z) and Tracheostomy (0B110F4, 0B110Z4, or other relevant tracheostomy codes).

Secondary outcomes assessed in this study included: length of stay (LOS) in days, total hospital charges (TOTCHG), and in‐hospital mortality (DIED).

The following covariates were included in the analysis:

- Demographic factors: age (categorized into < 1, 1–4, 5–12, 13–17, and 18–20 years), sex, race/ethnicity, and insurance type (public, private, other).
- Hospital characteristics: region and teaching status.
- Comorbidities: Identified via ICD codes in secondary diagnosis fields, these included asthma (J45.x), urticaria (L50.x), anaphylaxis (T78.2x), and drug allergies (T88.6x).

Asthma was included as a comorbidity of interest given its frequent clinical overlap with allergic conditions [10].

Descriptive statistics were used to summarize the study population, presenting frequencies and proportions for categorical variables, and means or medians for continuous variables. Comparative analyses were conducted to examine differences in demographics, comorbidities, hospital characteristics, and secondary outcomes (LOS, total charges, mortality) between angioedema patients who underwent airway intervention compared to those who did not. Chi‐square and Fisher's exact tests were used for categorical variables, and Wilcoxon rank‐sum tests were used for continuous variables.

Multivariable logistic regression was utilized to identify independent predictors of any airway intervention (yes/no), with age, sex, race, insurance, comorbidities, and hospital characteristics included as independent variables. Adjusted odds ratios (AORs) with 95% confidence intervals (CIs) were reported. For secondary outcomes, multivariable linear regression was used for LOS and log‐transformed total charges, with adjustments for relevant covariates. All analyses accounted for the complex survey design of KID using the survey package in R (svydesign and svyglm functions) implemented in R version 4.5.0 in RStudio [11]. Statistical significance was set at *p* < 0.05.

## 3 Results

The study identified a total of 4238 paediatric inpatient encounters with a primary or secondary diagnosis of angioedema from KID. Of these, 174 (4.1%) underwent airway procedures, including intubation or tracheostomy. The cohort was racially and regionally diverse: 50.3% of patients were White, 25.9% Black, 15.4% Hispanic, and 8.3% identified as Asian/Pacific Islander, Native American, or other/unknown race. Geographically, admissions were most common in the South (32.4%), followed by the Midwest (25.5%), West (22.2%), and Northeast (19.9%). The majority of hospitalizations occurred at urban teaching hospitals (62.8%), with additional cases at urban non‐teaching (26.0%) and rural hospitals (8.5%).

Descriptive comparisons revealed significant differences (Table 1).

**TABLE 1:** Descriptive comparison of paediatric angioedema hospitalizations with and without airway intervention.

Variable	Airway intervention (*n* = 174)	Non‐intervention (*n* = 4064)	*p*
*Demographics*			
Median age	16 years	11 years	**0.014**
Male	47.7%	47.6%	0.99
Female	52.3%	51.5%	0.99
*Hospital course*			
Median charges	$59 571	$7628	**< 0.001**
Median length of stay	6 days	2 days	**< 0.001**
Mortality rate	5.8%	0.15%	**< 0.001** (Fisher's)
*Comorbidities*			
Asthma	50.0%	90.3%	**< 0.001**
Anaphylaxis	31.2%	62.0%	**< 0.001**
Urticaria	5.4%	68.8%	**< 0.001**
Immune disorder	10%	47.6%	**< 0.001**
Drug allergy	0%	7.9%	NS (Fisher's 0.11)

*Note:* Bold values indicate statistical significance (*p* < 0.05).

Demographic analysis produced significant results when comparing groups. Patients requiring airway intervention were significantly older, with a median age of 16 years compared to 11 years in the non‐intervention group (*p* = 0.014). They had significantly longer median LOS (6 vs. 2 days, *p* < 0.001) and higher charges ($59 571 vs. $7628, *p* < 0.001). Mortality was also significantly higher in this group (5.8% vs. 0.15%, *p* < 0.001).

Comorbidities were assessed using Fisher's exact test. Among these, we found that asthma (50.0% vs. 90.3%, *p* < 0.001), anaphylaxis (31.2% vs. 62.0%, *p* < 0.001), urticaria (5.4% vs. 68.8%, *p* < 0.001), and immune disorders (10% vs. 47.6%, *p* < 0.001) were less common in the intervention group. Presence of a drug allergy showed no significant difference between groups (0% vs. 7.9%, *p* = 0.11).

Univariate analysis as shown in Table 2 was similar for the distribution of race and hospital region between those with and without airway interventions (*p* = 0.598 and *p* = 0.90, respectively). Differences that were not apparent in unadjusted comparisons may emerge in multivariable models after adjustment for potential confounding variables, which may explain the statistically significant associations observed in the regression analysis. Further analysis was performed with multivariable logistic regression (Table 3) to identify independent predictors of airway intervention. After adjusting for demographics, comorbidities, and hospital characteristics, Black (adjusted odds ratio [AOR] = 5.23, 95% CI: 1.56–19.30) and Hispanic ethnicity (AOR = 7.83, 95% CI: 1.51–44.89) were significantly associated with higher odds of undergoing airway intervention. Hospitalizations in the Northeast (AOR = 0.10, 95% CI: 0.015–0.55, *p* = 0.012) and South (AOR = 0.13, 95% CI: 0.026–0.49, *p* = 0.005) had significantly lower odds of intervention compared to the Midwest. Hospital type, insurance status, comorbidities (including asthma, anaphylaxis, and urticaria), and all age groups were not statistically significant predictors of airway intervention in adjusted models.

**TABLE 2:** Comparison of airway intervention vs. non‐intervention paediatric angioedema hospitalizations by race and hospital region.

Variable	Airway Intervention (*n* = 174)	Non‐Intervention (*n* = 4064)	*p*
*Race*			
White	50.3%	51.1%	0.598
Black	25.9%	22.1%	
Hispanic	15.5%	19.1%	
Asian/Pacific Islander	2.1%	2.7%	
Native American	1.4%	0.6%	
Other/Unknown	4.9%	4.5%	
*Hospital region*			
Northeast	21.3%	19.9%	0.90
Midwest	27.0%	25.5%	
South	30.5%	32.4%	
West	21.3%	22.2%	

**TABLE 3:** Multivariable logistic regression identifying predictors of airway intervention among paediatric angioedema hospitalizations.

Variable	Adjusted odds ratio (AOR)	95% CI	*p*
*Age*			
1–4 years	0.41	0.08–1.84	0.25
5–12 years	0.80	0.17–3.70	0.77
13–17 years	0.19	0.02–1.09	0.079
18–20 years	0.28	0.05–1.33	0.12
*Insurance*			
Private	2.05	0.65–6.73	0.23
Self‐Pay	0.65	0.02–7.95	0.754
Other	1.37	0.21–9.20	0.74
*Comorbidities*			
Asthma	0.70	0.17–2.49	0.59
Anaphylaxis	2.49	0.26–26.5	0.42
Urticaria	0.59	0.02–7.73	0.72
*Race*			
Black	5.23	1.56–19.3	**0.009**
Hispanic	7.83	1.51–44.9	**0.016**
Asian/Pacific Islander	9.67	0.63–172	0.10
Native American	1.79	0.08–17.6	0.65
*Hospital region*			
Northeast	0.10	0.015–0.55	**0.012**
South	0.13	0.026–0.49	**0.005**
West	0.51	0.12–2.00	0.35
*Hospital teaching status*			
Urban non‐teaching	4.23	0.23–88.1	0.33
Urban teaching	1.15	0.23–6.82	0.87

*Note:* Bold values indicate statistical significance (*p* < 0.05). Midwest region and rural hospitals served as the reference categories. Estimates for some categories have wide CIs due to small sample sizes.

Multivariable linear regression models were also performed for length of stay and total charges. For length of stay (log‐transformed), significant predictors included age group 1–4 years (shorter LOS, *p* = 0.044) and asthma (shorter LOS, *p* < 0.001). In addition, the age group 18–20 years trended toward significance with shorter LOS (*p* = 0.061). For total hospital charges (log‐transformed), asthma remained a significant predictor of lower charges (*p* < 0.001), and self‐pay insurance was also significantly associated with lower charges (*p* = 0.034). A trend toward higher charges was observed for Hispanic patients (*p* = 0.058), although this did not reach statistical significance. No other demographic, insurance, or hospital‐level factors were significantly associated with LOS or charges in adjusted models.

### 3.1 Supplemental Analysis by Procedure Type

Among 174 patients who received airway procedures, 156 underwent intubation and 17 underwent tracheostomy. As shown in Table 4, tracheostomy patients exhibited the most severe clinical profiles, with a median length of stay of 27 days, median total charges of $290 025, and a mortality rate of 23.5%. In contrast, patients who underwent intubation had a median LOS of 6 days, charges of $51 421, and a mortality rate of 3.8%. These values were all markedly higher than those observed in the non‐intervention group (median LOS 2 days, charges $7628, mortality rate 0.15%).

**TABLE 4:** Patient characteristics and outcomes by procedure type (intubation, tracheostomy, no procedure).

Variable	Intubation (*n* = 156)	Tracheostomy (*n* = 17)	Non‐Intervention (*n* = 4064)	*p*
*Demographics*				
Median Age	16 years	16 years	11 years	**0.049**
*Hospital course*				
Median Charges	$51 421	$290 025	$7628	**< 0.001** (Kruskal–Wallis)
Median Length of Stay	6 days	27 days	2 days	**< 0.001** (Kruskal–Wallis)
Mortality Rate	3.85%	23.5%	0.15%	**< 0.001** (Fisher's)
*Comorbidities*				
Asthma	55.6%	30.0%	90.3%	**< 0.001** (Fisher's)
Anaphylaxis	39.5%	0.0%	62.0%	**< 0.001** (Fisher's)
Urticaria	7.41%	0.0%	68.8%	**< 0.001** (Fisher's)
Immune disorder	13.3%	0.0%	47.6%	**< 0.001** (Fisher's)
Drug allergy	0%	0%	7.9%	0.409 (Fisher's)

*Note:* Bold values indicate statistical significance (*p* < 0.05).

Statistical tests confirmed significant differences across procedure types for median age (*p* = 0.049, Kruskal‐Wallis), length of stay and charges (both *p* < 0.001, Kruskal‐Wallis), and mortality (Fisher's *p* < 0.001). Comorbidities such as asthma, anaphylaxis, urticaria, and immune disorders also differed significantly between groups, with tracheostomy patients consistently showing the lowest prevalence of each.

## 4 Discussion

This national study of paediatric angioedema hospitalizations provides insights into the burden and predictors of critical airway intervention. While airway interventions are relatively rare among paediatric angioedema hospitalizations (4.1%), their occurrence is a strong indicator of disease severity and is associated with longer stays, higher charges, and elevated mortality, underscoring the need for clinical vigilance and multidisciplinary care.

In our cohort, patients requiring intervention experienced threefold longer LOS, eightfold higher charges, and nearly 40‐fold higher mortality (5.8% vs. 0.15%). In the published literature, fatal attacks are noted to thankfully be rare in childhood, with only 3 of 70 deaths occurring in patients under age 21 in the largest published series. However, these estimates largely derive from hereditary angioedema cohorts and may not capture mortality among hospitalized paediatric angioedema cases with critical airway compromise. The mortality seen in our cohort likely reflects the impact of severe presentations requiring advanced airway management, as well as the positive effects of advancements in emergency care, diagnostic capabilities, and targeted therapies [3, 6]. While life‐threatening attacks are generally considered uncommon, the unique vulnerability of smaller airways in children means that when airway compromise occurs, timely intervention is paramount [12].

Supplemental analysis by procedure type showed a severity gradient. Among those who underwent airway intervention, patients receiving tracheostomy had the most severe clinical profiles, with a median length of stay of 27 days, median total charges of $290 025, and a mortality rate of 23.5%. Intubation represented the more common airway intervention and was also associated with elevated resource utilization, including a median LOS of 6 days, charges of $51 421, and a mortality rate of 3.8%. In contrast, the non‐intervention group had significantly lower median LOS (2 days), total charges ($7628), and mortality (0.15%). These differences were statistically significant for age, length of stay, charges, and mortality. The increased age observed in the intervention group further supports the notion of heightened disease severity; this could potentially be linked to factors such as hormonal changes after puberty influencing angioedema presentation. This observation aligns with literature on hereditary angioedema, where approximately 50% of symptom onset occurs in the first decade and 90% within the first two decades, with an average age of onset around 11 years, suggesting that older age may correlate with more severe disease patterns requiring intervention [13]. These patterns reinforce the conclusion that any form of airway intervention, particularly tracheostomy, is a marker of heightened disease severity and resource burden in paediatric angioedema.

Multivariable analysis revealed significant racial and ethnic disparities in the likelihood of undergoing airway intervention, with Black and Hispanic patients having higher adjusted odds compared to White patients. This finding aligns with established literature on racial disparities in healthcare access and outcomes, suggesting that systemic or institutional factors may influence the clinical decision‐making process for airway management in different patient populations [14]. Further investigation is needed to understand the underlying drivers of these disparities, which could include differences in angioedema severity, access to care, bias in clinical assessment, or variations in the timing of initial treatment.

Lower rates of urticaria, anaphylaxis, and asthma in the airway intervention group may reflect differences in underlying angioedema mechanisms. Histaminergic angioedema is frequently associated with urticaria and allergic comorbidities, whereas bradykinin‐mediated angioedema often presents with isolated swelling and limited response to antihistamines or epinephrine [7]. One possible explanation for the observed pattern is that cases requiring airway intervention may include a higher proportion of bradykinin‐mediated events, which are known to carry a greater risk of airway compromise [5]. However, because the ICD codes used in this dataset are broad and do not distinguish angioedema subtypes, this interpretation should be considered hypothesis‐generating rather than definitive. Further study is necessary to determine the utility of these patient factors in predicting the need for airway intervention.

In linear regression models of resource utilization, asthma emerged as a consistent predictor of decreased hospital burden as it was significantly associated with shorter length of stay and reduced total charges. This further strengthens the argument that angioedema in asthmatics may be more likely to be histaminergic and thereby responsive to standard treatments. In addition, self‐pay insurance was associated with lower hospital charges, while Hispanic ethnicity showed a trend toward higher charges. These findings suggest that both clinical and socioeconomic factors may influence resource utilization in paediatric angioedema hospitalizations, though the degree of missing data in these models limits definitive conclusions.

Another interesting finding is the association between hospital regions and the odds of airway intervention. Patients in the Northeast and South had significantly lower odds of undergoing a procedure compared to those in the Midwest. The Midwest served as the reference region in adjusted models as it had the highest proportion of airway intervention cases, allowing for clearer comparisons across regions with lower intervention rates. This difference in odds could be due to variations in regional practice patterns, differences in patient populations, or variations in the availability of subspecialty care. It highlights the potential for geographic variation in the management of paediatric angioedema and warrants further research to understand the clinical and systemic factors contributing to these regional differences.

This study's findings have important implications for clinical practice. The identification of racial and ethnic groups with higher odds of intervention provides a basis for targeted clinical vigilance. Although age was not a statistically significant predictor in adjusted models, clinical awareness may still vary across age groups given the differences observed in descriptive comparisons. The inverse association with comorbidities like asthma and urticaria provides a crucial clinical clue for distinguishing angioedema subtypes and guiding treatment decisions. The study underscores the need for early recognition, prompt referral, and interdisciplinary care to improve outcomes in paediatric angioedema.

A strength of this study is its utilization of KID, which provides a large, national, all payer sample of paediatric hospitalizations across the United States. This enables the generation of national estimates and the analysis of relatively rare but critical conditions like paediatric angioedema requiring airway intervention. Such scenarios would be difficult to study using smaller, single centre cohorts. However, several limitations stem from its retrospective, cross sectional design and reliance on administrative data. Additionally, several regression estimates demonstrated wide confidence intervals due to small subgroup sizes and the relatively rare occurrence of airway interventions. As a result, estimates for some demographic categories should be interpreted with caution, as limited statistical precision may affect the stability of these models. Notably, the ICD codes used to identify angioedema, such as T78.3, are broad in scope. This code encompasses allergic angioedema, giant urticaria, and Quincke's edema, while explicitly excluding general urticaria (L50 codes), which may introduce ambiguity in distinguishing between mast cell‐mediated and bradykinin‐mediated etiologies. Variability in clinical documentation and coding practices, such as whether a case is labelled as “giant urticaria” versus standalone angioedema, can lead to misclassification bias and may influence the observed associations with comorbidities. Furthermore, administrative datasets like KID are primarily collected for billing rather than clinical documentation and therefore lack detailed measures of disease severity. Important clinical variables such as laryngoscopic findings, degree of airway swelling, timing of therapeutic interventions, and medications administered during hospitalization are not available within the database. As a result, it is not possible to determine whether differences in airway intervention rates reflect underlying differences in disease severity or variations in clinical management and institutional practice patterns. These limitations must be considered when interpreting the findings, particularly with respect to angioedema subtypes and inferred pathophysiology.

Furthermore, the cross‐sectional nature of the data prevents the establishment of causal relationships; the identified “predictors” should be interpreted as associations. The triennial data collection of the KID, coupled with the absence of data for 2015 due to the ICD‐9 to ICD‐10 coding transition, means that continuous annual trends cannot be precisely assessed, and comparisons across the transition period require careful interpretation. Finally, the exploratory linear regression models for length of stay and hospital charges were limited by missing data within certain variables included in the adjusted models. This reduced the effective sample size available for these analyses and may limit the generalizability and statistical power of the findings related to resource utilization. Additionally, hospital charges were analysed as reported in the KID database and were not adjusted for inflation across the study period (2003–2019). As a result, comparisons of hospital charges across different years should be interpreted cautiously. Future research should aim to overcome the limitations of administrative data by incorporating more granular clinical information, potentially through prospective studies or detailed chart reviews, to better characterize angioedema aetiologies and their progression in paediatric patients. Developing more specific ICD codes or supplementary clinical documentation guidelines for angioedema subtypes would enhance the accuracy of large database studies. Further investigation into the “protective” associations observed with urticaria and asthma could involve examining specific angioedema subtypes within these patient populations, potentially revealing differential responses to treatment or underlying pathophysiological mechanisms. Longitudinal studies are needed to understand the long‐term outcomes and recurrence patterns in paediatric angioedema, particularly for those requiring airway interventions. Additionally, research focusing on early identification and targeted intervention strategies for high‐risk paediatric populations is crucial to reduce morbidity and mortality associated with critical airway compromise.

## 5 Conclusion

Paediatric angioedema hospitalizations requiring airway interventions, though infrequent, are consistently associated with a significantly higher burden of disease, marked by prolonged hospital stays, elevated healthcare costs, and a dramatically increased risk of mortality. The inverse association between urticaria, anaphylaxis, and asthma and the need for airway intervention suggests that angioedema aetiology plays a critical role in determining disease severity and responsiveness to treatment. The observed racial/ethnic and geographic disparities in intervention rates highlight the need for further investigation into systemic, institutional, and clinical drivers of airway management decisions in paediatric angioedema.

## Author Contributions

S.T.: Conceptualization, Data curation, Formal analysis, Writing – original draft. A.M.: Conceptualization, Writing – original draft. G.N.P.: Supervision, Writing – review and editing. B.K.R.: Supervision, Writing – review and editing. R.K.: Conceptualization, Supervision, Writing – review and editing.

## Funding

The authors have nothing to report.

## Ethics Statement

This study was reviewed by the Rutgers Institutional Review Board and determined to be exempt from IRB oversight, as it involved secondary analysis of de‐identified, publicly available data from the Healthcare Cost and Utilization Project (HCUP) Kids' Inpatient Database (KID). No direct interaction with human subjects or access to identifiable private information occurred.

## Consent

The authors have nothing to report.

## Conflicts of Interest

The authors declare no conflicts of interest.

## Data Availability

The data that support the findings of this study are available from the Healthcare Cost and Utilization Project (HCUP) Kids' Inpatient Database (KID) maintained by the Agency for Healthcare Research and Quality (AHRQ). Restrictions apply to the availability of these data, which were used under licence for this study. Data are available at https://hcup‐us.ahrq.gov/kidoverview.jsp with the permission of HCUP.
